# Understanding the Immunological Quality of Breast Milk in Maternal Overweight and Obesity

**DOI:** 10.3390/nu15245016

**Published:** 2023-12-05

**Authors:** Anita Froń, Magdalena Orczyk-Pawiłowicz

**Affiliations:** Division of Chemistry and Immunochemistry, Department of Biochemistry and Immunochemistry, Wroclaw Medical University, M. Skłodowskiej-Curie 48/50, 50-369 Wroclaw, Poland

**Keywords:** breast milk, maternal obesity, breastfeeding, immune properties, metabolic disorders, BMI

## Abstract

Maternal obesity, affecting many pregnant women globally, not only poses immediate health risks but also modulates breast milk composition. Obesity is linked to inflammation and oxidative stress, impacting breast milk’s immune properties. This paper explores the intricate relationship between maternal metabolic disorders, such as obesity, and breast milk’s immunological components. We conducted a thorough search for original and review articles published until 17 October 2023 in the PUBMED/Scopus database. This search included several terms related to human breast milk, immunological properties, and obesity. Articles were selected with the consensus of all authors. Maternal metabolic disorders have discernible effects on the composition of immune-related components in breast milk, such as immunoglobulins, lactoferrin, leptin, ghrelin, adiponectin, C-reactive protein, growth factors, extracellular vesicles, and lymphocytes. These changes in breast milk composition can significantly impact the newborn’s immune system, with potential long-term health implications beyond the immediate postnatal period. Maternal metabolic health is a critical factor in shaping the health trajectory of the neonate through breastfeeding, although the full advantages of breastfeeding for children of mothers with obesity remain uncertain. Ongoing research aims to understand and unravel these links.

## 1. Introduction

Newborns enter the world with an underdeveloped immune system. The innate immune system acts as the first line of defense against pathogens and starts to build nonspecific memory as a response to pathogen exposure, particularly following the transition of neonates from a sterile uterine environment to a microbe-rich external world [1,2,3]. The World Health Organization advises exclusive breastfeeding for the initial six months of an infant’s life. Subsequently, as infants’ nutritional needs evolve, it is recommended to introduce nutritionally sufficient and safe complementary foods while continuing breastfeeding for a duration extending up to two years or beyond [4]. A similar position, endorsing breastfeeding, was adopted by the “Academy of Nutrition and Dietetics” [5]. Infant feeding, as an integral aspect of the developmental origins of health and disease (DOHaD), emphasizes the importance of nutrition and various environmental factors during the perinatal period for the development of a child’s health [6,7]. Breastfeeding yields numerous short-term and long-term health benefits. Multiple studies confirm that breastfed children face lower incidences of both acute and chronic illnesses. Exclusive breastfeeding for the first six months of life significantly reduces mortality rates from infectious diseases and overall mortality risk [8]. Breastfed infants experience lower rates of gastrointestinal tract infections, necrotizing enterocolitis (NEC), respiratory infections, and sudden infant death syndrome. Moreover, breastfeeding is linked to decreased risks of chronic conditions such as allergies, asthma, diabetes, obesity, irritable bowel syndrome, and Crohn’s disease throughout childhood and adulthood. Enhanced cognitive development is also associated with prolonged and exclusive breastfeeding [9,10,11,12,13,14].

In recent years, maternal obesity has become a burgeoning global health concern, affecting a significant proportion of expectant mothers [15]. It is estimated that in the United States, one in two pregnant women is overweight [16]. Obesity during pregnancy is known to exert profound effects on both maternal and fetal health, with implications that extend to the postnatal period. Beyond the immediate metabolic and cardiovascular risks posed to the mother, emerging research has unveiled the intricate interplay between maternal adiposity and the composition of breast milk (BM) [17,18,19]. It is also important to note that obesity is associated with reduced milk production in mothers [20,21]. Obesity is associated with an increased presence of proinflammatory cells like macrophages in adipose tissue, contributing to chronic inflammation. These immune cells produce and release proinflammatory molecules such as TNF-alpha, IL-6, and IL-1b, leading to an inflammatory state. In obesity, excess calorie intake triggers oxidative stress within cells, causing an overflow of reactive oxygen species. Larger adipose depots also reduce the body’s antioxidant capacity. Additionally, hypertrophic adipocytes can rupture, releasing internal contents that further induce inflammation [22,23,24,25,26].

The mammary gland undergoes significant changes throughout a woman’s life. Initially, it consists of branched ducts that invade the surrounding adipose tissue. During puberty, hormonal stimulation causes these ducts to grow and branch, increasing the gland’s volume. In pregnancy and lactation, the gland’s epithelial cells proliferate and transform into milk-secreting alveolar cells [27,28]. Breast milk is a complex fluid composed of various components with distinct origins. Some components are exclusively produced by the specialized mammary secretory cells, including lactose, casein, and lactalbumin, while others are acquired from the bloodstream, such as immunoglobulins and leukocytes. Adipokines, however, can originate from either of these two sources [29,30,31,32,33]. This implies that alterations in the mother’s body can result in modifications to the composition of her breast milk.

Moreover, mothers with obesity have an increased risk of the following outcomes: gestational hypertension, preeclampsia, and gestational diabetes [34,35]. Gestational diabetes mellitus (GDM) is becoming increasingly prevalent worldwide and is among the most common medical complications during pregnancy [36]. It poses significant health risks for both the mother and the developing fetus, both in the short term and long term. These risks include a higher likelihood of the mother developing type 2 diabetes (T2DM) in the future and potential adverse cardiometabolic effects on the offspring [37]. Birth complications associated with GDM often involve a higher risk of cesarean delivery, shoulder dystocia, and birth injuries, often due to the fetus being larger in size [38].

Hypertensive disorders during pregnancy exert significant impacts on the well-being of mothers, fetuses, and newborns [39]. Among these conditions, preeclampsia is a prevalent pregnancy-specific disease characterized by hypertension and multiple organ dysfunctions, including issues with the kidneys, liver, and lungs [40]. Importantly, women with a history of preeclampsia face a 2- to 5-fold increased risk of developing cardiovascular diseases, which account for a substantial portion of female mortality worldwide [41]. For the fetus, the most significant risks associated with hypertensive disorders during pregnancy include premature birth and intrauterine growth restriction (IUGR) [39]. Hypertensive disorders during pregnancy affect breastfeeding practices. Women with these conditions breastfed for approximately 6.26 weeks less during the year following childbirth and were significantly more likely to report having an insufficient milk supply [42].

This scientific paper delves into the intricate interplay between maternal metabolic disorders and the immunological quality of breast milk. It explores the alterations in key immunological components, such as immunoglobulins, cytokines, and immune cells, which are integral to the infant’s immune defense mechanisms. Moreover, it investigates the potential implications of these immunological changes for the infant’s susceptibility to infections, as well as their long-term health outcomes, including the risk of obesity and metabolic disorders.

As we embark on this exploration, it is imperative to recognize the broader significance of unraveling the complexities surrounding breast milk composition in the context of maternal metabolic disorders. By gaining a deeper understanding of how maternal metabolic disorders impact the immunological properties of breast milk, we may uncover opportunities for intervention and support to mitigate potential risks to the health and well-being of the next generation.

## 2. Materials and Methods

For this narrative review, we conducted comprehensive English-language literature research for original and review articles published until 17 October 2023 in the PUBMED/Scopus database. We searched for the following terms, alone or in combination: human milk, breast milk, breastfeeding, lactation, milk composition, body mass index (BMI), maternal obesity, maternal overweight, immunological properties, metabolic hormones, macronutrients, carbohydrates, lactose, human milk oligosaccharides (HMOs), lipids, Igs, secretory IgA (SIgA), IgA, IgG, IgM, lactoferrin, growth factors, vascular endothelial growth factor (VEGF), hepatic growth factor (HGF), epidermal growth factor (EGF), insulin-like growth factors (IGF-1), heparin-binding epidermal growth factor (HB-EGF), transforming growth factor-β2 (TGF-β2), adipokines, cytokines, interleukins (ILs), tumor necrosis factors (TNFs), interferons (IFNs), adiponectin, ghrelin, leptin, exosomes, microbiome, microbiota, casein, free amino acids (FAAs), branched-chain amino acids (BCAA), polyamines, nucleotides, osteopontin, C-reactive protein (CRP), mRNA, microRNA (miRNA), leukocytes, B-lymphocytes, lysozyme, α-lactalbumin, lactoperoxidase, and haptocorrin. The selection of immunological factors for analysis was based on the most recent scientific publications addressing this topic [8,43,44,45]. We found 342 related articles. The relevant studies were identified by evaluating the abstracts, and complete articles were obtained in cases where abstracts were unavailable. Duplicate papers were removed, and the data were screened to exclude irrelevant works. Case reports, comments, conference papers, commentaries, surveys, and animal studies were all excluded from the full-text publications. Additional manual searches were conducted on the indicated bibliographies, taking into consideration the articles’ novelty, quality, and clinical significance. After applying the exclusion criteria, 169 full-text manuscripts were assessed for eligibility with the consensus of the authors.

## 3. Components of Breast Milk

The composition of human milk undergoes a transition from colostrum to transitional milk and finally to mature milk as lactation progresses. Colostrum, the initial milk produced by mothers after giving birth, is present in small amounts during the first two to four days. Transitional milk follows, characterized by increased milk production from the fifth day to around two weeks after childbirth, and it shares similar characteristics with colostrum. Approximately two weeks postpartum, human milk reaches its mature stage [46].

During each breastfeeding session, the initial milk expressed (foremilk) is more watery and contains a higher proportion of lactose, which quenches the baby’s thirst. Subsequently, the hindmilk that follows is thicker and richer in fat, providing the necessary nutrients for the baby’s requirements [47].

### 3.1. Proteins

The proteins present in breast milk serve a diverse array of purposes: enhancing micronutrient bioavailability, aiding in immunological defense, promoting intestinal growth, influencing the microbiome, and boosting cognitive functions [45,48]. Human milk primarily contains two main proteins: casein and whey. When consumed, casein coagulates into clots or curds within the stomach, whereas whey remains in liquid form, making it more readily digestible. The proportion of whey protein in breast milk varies based on the milk’s stage, ranging from 80% in early lactation to 50% in later stages. The whey-to-casein ratio in human milk changes from approximately 70/30 to 80/20 during the initial phases of lactation, gradually approaching a balanced 50/50 ratio as lactation progresses [47]. In the early stages following the infant’s birth, the protein content is notably high, ranging from 14 to 16 g/L. However, as the infant reaches 3 to 4 months of age, this protein content gradually decreases to a range of 8 to 10 g/L. After the 6-month mark, the protein content undergoes a further reduction, reaching a level of approximately 7 to 8 g/L [8].

Nevertheless, there is no difference in total protein concentration in breast milk between overweight/obese and normal-weight women regardless of lactation stage, as indicated by multiple studies [49,50,51,52].

### 3.2. Carbohydrates

Breast milk contains a significant proportion of carbohydrates, typically ranging from 60 to 70 g/L, contributing to approximately 40% of the total caloric content, with lactose being the predominant carbohydrate component [8]. The lactose content in breast milk shows variations depending on the stage of lactation. It ranges from 45 to 64.7 g/L in colostrum, 48 to 73.7 g/L in transitional milk, and 67.8 to 77 g/L in mature milk [53].

Compared to women with normal weight, overweight and obese women had higher lactose concentrations in colostrum, with a mean difference of 2.24 g/L [54]. However, no significant differences in lactose concentration were found in either transitional or mature breast milk samples between overweight/obese and normal-weight women [54]. In contrast, colostrum glucose concentrations were higher in obese and diabetic mothers [55,56,57].

Human milk oligosaccharides (HMOs) play a vital role in the carbohydrate composition of maternal milk. In mature milk, the average HMO content stands at 12.9 g/L, increasing to 20.9 g/L four days after childbirth. Each HMO structure comprises a range of saccharide units, varying from 3 to 22 molecules, and consists of a combination of five distinct sugars: L-fucose, D-glucose, D-galactose, *N*-acetylglucosamine, and *N*-acetylneuraminic acid. The spectrum of HMO diversity is extensive, including over 200 unique varieties present in human milk. Notably, all these varieties share a common feature: lactose situated at their reducing end [45].

By functioning as prebiotics and metabolic substrates, HMOs actively promote the growth of beneficial commensal bacteria while simultaneously inhibiting the growth of potentially harmful microorganisms. HMOs also possess distinctive antimicrobial properties, targeting specific pathogens like *Streptococcus pneumoniae*, *E. coli*, Group B *Streptococcus*, and *Campylobacter* among others, through the inhibition of pathogen adhesion to the intestinal epithelium [8,45,58].

Beyond their contributions to microbiota modulation, HMOs exert influence on intestinal epithelial cells, triggering various responses. They regulate cellular processes, including growth, differentiation, and apoptosis. Additionally, HMOs have the potential to influence immune responses, potentially shifting T-cell responses to achieve balanced Th1/Th2-cytokine production. The multifaceted and intricate roles played by HMOs underscore their importance in reinforcing neonatal gastrointestinal robustness and augmenting immune defense mechanisms [8,45,58].

Noteworthy findings reveal that levels of 2’-fucosyllactose (2′-FL) were significantly lower in BM from overweight and obese mothers at 1 month of lactation. This decrease could potentially contribute to lower infant weight, height, and growth, as well as a reduced level of protection against infections [59]. Opposite results were obtained by Lagstrom et al. [60] and Wang et al. [61]. Lagstrom’s study revealed a significant correlation between 2′-FL levels and maternal pre-pregnancy BMI at the 3-month point of breastfeeding, while Wang’s research found this correlation in breast milk samples collected one month after lactation. Additionally, the content of lacto-*N*-fucopentaose I (LNFPI) was significantly reduced in BM from overweight and obese mothers at 1 month of lactation. This reduction may compromise protection against infections and negatively affect neonatal gut microbiota, particularly by reducing *Lactobacillus* spp. On the other hand, lacto-*N*-fucopentaose II or III (LNFPII/III) exhibited a 1674-fold increase [59]. Furthermore, higher maternal BMI is also associated with lower levels of acidic HMOs, suggesting that maternal adiposity might affect HMOs sialylation. On the other hand, levels of lacto-*N*-neotetraose, 3-fucosyllactose, 3-sialyllactose, and 6-sialyllactose were increased at two months of lactation in mothers with obesity [62].

Beyond lactose and HMOs, maternal factors can also impact the levels of various components in breast milk, including certain monosaccharides, which play a role in influencing the infant’s immune system. For example, arabinose serves as a carbon source for bacteria and may impact the activity of certain pathogens by potentially reducing their virulence. Arabinose levels were 1.72 times higher in BM from overweight and obese mothers at 6 months of lactation. Additionally, glucose-6-phosphate (G6P) is known to be involved in shielding against oxidative stress and could potentially serve as an energy source for infants. G6P levels were 2.07 times higher in the BM of overweight mothers at 6 months [59].

### 3.3. Fats

Fat, constituting approximately 38–39 g/L, represents nearly half of the infant’s nutritional intake from BM. Triacylglycerides are the predominant lipid component, making up approximately 98% of the overall lipid content. The remaining fraction consists primarily of diacylglycerides, monoacylglycerides, free fatty acids (FFAs), phospholipids (PLs), and cholesterol. The most abundant fatty acids in breast milk—oleic, palmitic, and linoleic acid—are typically found at the sn-1, sn-2, and sn-3 positions, respectively. Moreover, breast milk contains long-chain polyunsaturated fatty acids (LCPUFAs), which serve as specialized pro-resolving mediators and play a crucial role in supporting growth, organ development, and enhancing immune maturation. Additionally, these compounds exhibit anti-inflammatory properties and have demonstrated potential benefits in conditions such as inflammatory bowel disease, which shares pathological similarities with necrotizing enterocolitis (NEC). Furthermore, their presence in breast milk is associated with a reduced risk of allergic diseases and respiratory illnesses during the early years of a child’s life [8,45,63,64,65,66].

PLs are primarily localized within the milk fat globule membrane, coexisting with cholesterol, enzymes, glycolipids, and glycoproteins [67]. PLs exhibit a diverse distribution among five major classes. The predominant compounds include phosphatidylethanolamine (PE) at a proportion of 29% to 33%, phosphatidylcholine (PC) ranging from 20% to 25%, and sphingomyelin (SM) comprising 22% to 29%, while phosphatidylinositol (PI) and phosphatidylserine (PS) represent minor components [68,69,70]. The total PLs content in human milk varies between 9.8 and 47.4 mg per 100 g. Notably, a higher concentration of total phospholipids is observed in colostrum and in the later stages of lactation (post 200 days postpartum) compared to samples collected between 10 and 45 days postpartum [69,71,72]. PC and SM play a crucial role as major sources of choline, collectively contributing to approximately 40–50% of cellular membrane composition. Choline, a precursor to the neurotransmitter acetylcholine, holds significance in regulating signal transduction and serves as a methyl group source in intermediate metabolism, essential for optimal brain development [73,74,75]. Furthermore, sphingomyelin is instrumental in driving this process, not only through the regulation of nuclear activity but also by actively engaging in the formation of myelin sheets. This dual mechanism is thought to be a fundamental element in fostering the maturation of nerve cells [76]. Phospholipids have been observed to impact the downregulation of stearoyl-CoA desaturase-1 (SCD1) expression, resulting in a decreased presence of triacylglycerol and cholesterol in both the liver and the bloodstream [77].

The presence of short-chain fatty acids (SCFA) in BM carries significance not only as an energy source but also for promoting the healthy development of the gastrointestinal tract. Additionally, it has been demonstrated that the lipids present in breast milk can deactivate several pathogens under laboratory conditions, including Group B *streptococcus* (GBS) [45,78].

Mothers with obesity tend to have higher fat and energy content in breast milk. Specifically, there is no disparity in fat concentration between overweight/obese and normal-weight women in colostrum. Yet, during the transitional milk phase, overweight/obese mothers exhibit lower fat concentration than their normal-weight counterparts. However, in mature milk, overweight/obese mothers have a higher fat concentration compared to those with normal weight [54,79]. Surprisingly, research by Dritsakou et al. [80] found that in overweight/obese women, higher levels of fat were observed regardless of lactation stage. These findings were substantiated by the research conducted by Han and colleagues [81].

## 4. Breast Milk Components Affecting Immunity

The body’s defense mechanisms against invading pathogens involve both the innate and adaptive immune systems. The innate immune system relies on general, non-specific defenses like physical barriers, secreted molecules, and various types of cells. Breast milk contains a range of these innate immune components, including fatty acids, lysozyme, lactoferrin, lactalbumin, lactoperoxidase, casein, human milk oligosaccharides (HMOs), nucleic acids, cytokines, and cytokine receptors. These non-immunoglobulin products offer initial protection at mucosal surfaces, especially when infants lack sufficient antibody levels to combat specific pathogens [82,83,84,85,86].

On the other hand, the adaptive immune system consists of specialized components, such as immunoglobulins like IgM, IgG, and IgA, with secretory IgA (SIgA) accounting for roughly 90% of these. They stand out as one of the earliest protective elements identified in human milk. The presence of lymphocytes further contributes to the body’s adaptive immune responses [82,83,85,86].

Changes and overarching trends in the immunological properties of breast milk among obese women are presented in a condensed form in Table 1.

### 4.1. Immunoglobulins

The presence of immunoglobulins (Igs) in BM plays a crucial role in providing immune protection to infants. In essence, these immunoglobulins, particularly SIgA, are fundamental in safeguarding newborns against pathogens by preventing adherence and supporting local immunity. Other immunoglobulins, such as IgM, IgG, and their subclasses, also contribute to immune defense and regulation in neonates.

Secretory IgA stands out as the most abundant, accounting for 80–90% of the total Ig content. Of approximately 0.5 to 1.0 g/day of SIgA ingested by exclusively breastfed infants, only 10% is absorbed by the intestine and transferred to the bloodstream. Colostrum contains higher concentrations of SIgA (around 12 g/L) compared to mature milk (~1 g/L), highlighting its protective role. SIgA’s primary function is to bolster local immunity in the newborn by preventing the adherence of pathogens to the intestinal mucosal surface, neutralizing toxins, as well as inducing tolerance to microbial and food antigens. Breast milk harbors SIgA antibodies specific to pathogens like Vibrio cholerae, Campylobacter, and respiratory tract infections. The presence of SIgA in human milk acts as a protective factor, particularly beneficial for premature infants, as it defends against infections and reduces the risk of NEC [45,120,121,122].

Immunoglobulin M is the second most abundant Ig in colostrum, and high avidity IgM antibodies reactive with viruses and bacteria contribute to protecting mucosal surfaces in infants. The IgM content exhibits a decreasing trend, with a decline from colostrum (~600 mg/L) to transition milk (~430 mg/L), and ultimately to mature milk (~260 mg/L). IgG, found at lower concentrations in milk, exhibits neutralizing and opsonizing activities. There have been no standardized studies to date that unequivocally determine IgG levels. However, several studies have indicated that their concentrations typically vary between 5 and 1100 mg/L [123,124,125,126,127]. IgG1 and IgG3, subclasses of IgG, activate the complement pathway for pathogen clearance. IgG4 increases the allergen response and possesses anti-inflammatory properties, while IgG2 is associated with defense against bacterial antigens and may play a role in the Th1 immune response. Additionally, other Igs like IgE and IgD are present in breast milk, although their roles and effects in neonates are less understood [128].

When analyzing lactation in relation to Igs, significant differences were observed among different groups of puerperal patients. Women with obesity exhibited altered levels of various immunoglobulin classes in their breast milk compared to those with normal body weight. Specifically, the content of IgG was 2.1 times lower, IgM was 1.9 times lower, and IgA was 2.2 times lower in the breast milk of obese mothers compared to mothers with a normal BMI [87]. In contrast, Fujimori et al. [49] reported significantly elevated concentrations of SIgA in colostrum and IgA in serum among overweight and obese mothers (*p* = 0.001). The reasons why obesity leads to higher SIgA concentrations are not fully understood. However, this may be linked to chronic low-grade inflammation, characterized by increased levels of the pro-inflammatory marker IL-6 in the bloodstream [49].

### 4.2. Lactoferrin

Lactoferrin, found in human breast milk, exhibits varying concentrations, ranging from around 7 g/L in colostrum to 1 g/L in mature milk. As a nonheme iron-binding protein, lactoferrin exerts antimicrobial effects by targeting iron availability. It possesses both bacteriostatic and bactericidal activities against pathogens dependent on iron. Furthermore, lactoferrin promotes the growth of bacteria with low iron requirements, which are considered beneficial for human health, including *Lactobacillus* and *Bifidobacterium* [8,129]. Recent evidence highlights the potential of lactoferrin to significantly reduce the incidence of serious complications in premature infants, such as NEC and late-onset sepsis (LOS), while also lowering the risk of infection-related mortality [130]. Lactoferrin contributes to antibacterial and antiviral actions in the intestinal tract by directly affecting pathogens and influencing gastrointestinal and immune functions through receptor-mediated uptake and gene transcription modulation. Moreover, it stimulates macrophage activation, aiding the clearance of Gram-positive bacteria. It also upregulates the expression of polysialic acid, a marker associated with neuroplasticity, cell migration, and differentiation. Additionally, lactoferrin enhances phosphorylation of CREB, a crucial protein in neurodevelopment and cognition [131].

Interestingly, the level of lactoferrin was notably higher in the BM of mothers who exceeded the 90% weight for height (WFH), an indicator often used to assess obesity, which is equivalent to a BMI greater than 30 kg/m². Within the first 15 days after childbirth, lactoferrin concentration in the breast milk of Aboriginal mothers was 3.49 mg/mL for those with a WFH greater than 90% and 2.89 mg/mL for those with a WFH less than 90%. After 15 days postpartum, the concentrations dropped to 1.23 mg/mL and 0.87 mg/mL, respectively. Similarly, for Caucasian women, 15 days postpartum, lactoferrin concentration was 1.42 mg/mL for those with a WFH greater than 90% and 0.66 mg/mL for those with a WFH less than 90% [88]. Contrarily, Dyndar et al. [87] arrived at a differing conclusion. Their research suggested that the level of lactoferrin in the breast milk of mothers with obesity was 1.6 times lower compared to those with a normal BMI.

### 4.3. Cytokines

Breast milk contains a variety of cytokines that play crucial roles in the immature immune system development of newborns. These cytokines include tumor necrosis factors (TNFs), such as TNF-α, interleukins (IL), including IL-1β, IL-6, IL-8, and IL-10, and interferons (IFNs), specifically interferon-γ. They contribute to immunomodulation and passive protection, reducing the risk of infections. The presence of cytokines is particularly significant for newborns, who are generally deficient in these proteins [8,58,132,133].

Among the most abundant cytokines in BM is IL-6, which is associated with inflammation and fever. IL-6 plays a role in neutrophil recruitment, intestinal development, and protection against TNF-α mediated damage. IL-2 stimulates T lymphocyte and natural killer cell growth, influencing immune system development and Th1/Th2 differentiation. IL-8 recruits leukocytes and their flow from the mother’s circulation into her milk, while interleukin-4 participates in allergic reactions. The anti-inflammatory cytokine IL-10 inhibits macrophages, T-cells, and natural killer cell development, enhancing B-cell differentiation for immunoglobulin synthesis. IL-10 helps modulate cytokine responses to infection, balancing immune defense and minimizing tissue damage [132,134].

Cytokines in colostrum stimulate IL-1, IL-3, and IL-6 release from peripheral blood mononuclear cells (PBMC) in infants. IL-1β activates TNF-α secretion, promoting macrophage activity and cellular immunity. TNF-α increases intestinal permeability, and IFN-γ potentiates its effects. IL-2, which stimulates B- and T-cell proliferation and other pro-inflammatory cytokines, is also influenced by colostrum levels. Colostrum’s inhibitory effects on NK cell activity can be reversed by recombinant IL-2 [132,135]. BM’s cytokines contribute significantly to the immune system development and defense in newborns, influencing various immune responses and maintaining a delicate balance between pro-inflammatory and anti-inflammatory effects.

Research revealed that the IL-6 concentration in colostrum was notably higher in overweight and obese mothers (81.8 pg/mL) compared to mothers with a healthy BMI (62.9 pg/mL). However, the scenario shifted in the case of 1-month milk, where the IL-6 concentration for overweight and obese mothers (13.2 pg/mL) was lower than that for mothers with a normal BMI (22.1 pg/mL) [89]. Such fluctuations could potentially influence infant growth and body composition [103]. Similarly, the trend also holds true for IL-10 and IL-4. In the colostrum of lean mothers, its concentration is 8.8 pg/mL and 18.2 pg/mL, while in overweight mothers, it is 11.3 pg/mL and 20.8 pg/mL, respectively [89]. One study reported TNFα concentrations of 11.4 pg/mL in colostrum and 10.2 pg/mL in 1-month milk for mothers with a BMI > 25 kg/m^2^. These values were contrasted with the data from mothers with a BMI ≤ 25 kg/m^2^, for whom TNF-α concentrations are expected to be around 9.9 and 10.6 pg/mL, respectively [89].

### 4.4. Growth Factors

Growth factors present in BM play a crucial role in facilitating the maturation of the intestinal mucosal barrier, offering both growth-promoting and protective effects to the neonatal immature gastrointestinal tract. Notably, vascular endothelial growth factor (VEGF), hepatic growth factor (HGF), and epidermal growth factor (EGF) stand out as key contributors, with colostrum containing higher concentrations of these factors compared to late milk [134].

EGF is closely associated with neonatal intestinal development and plays a role in the response and repair of the intestinal lining following injury or infection. It is also suggested to influence goblet cells and the synthesis of mucin in the intestinal epithelium. VEGF, on the other hand, governs angiogenesis and vasculogenesis. Interestingly, the combined action of VEGF and HGF demonstrates superior potential for enhancing blood vessel formation within the neonatal intestinal tract while concurrently reducing inflammation and edema. Insulin-like growth factors (IGF-1) and heparin-binding epidermal growth factor (HB-EGF) share a common function in enhancing cell survival through the suppression of apoptosis. IGF-1, in particular, has been observed to stimulate the proliferation of intestinal stem cells, contributing to the overall health of the intestine.

Granulocyte colony-stimulating factor (G-CSF), a glycoprotein, plays a pivotal role in the proliferation and differentiation of granulocytes and neutrophils. Remarkably, G-CSF also contributes to maintaining the integrity of the gut barrier and the well-being of epithelial cells in the neonatal intestine [58,134,136,137].

Transforming growth factor-β2 (TGF-β2), abundant in human milk, holds significant immunomodulatory influence over intestinal maturation, immunoglobulin synthesis, and the suppression of T-cells. Breast milk with elevated TGF-β2 levels correlates with increased diversity in neonatal intestinal microbial composition, a factor known to reduce susceptibility to immunological disorders in adulthood [138,139,140].

Interesting findings were reported by Khodabakhshi [90]. His research revealed a significant correlation between a decrease in EGF levels in breast milk and higher infant body weight. Mothers of infants with obesity exhibited notably lower EGF levels (38 pg/mL) compared to mothers with normal-weight infants (40 pg/mL). This might be attributed to the positive correlation between EGF and ghrelin. It was noted that the concentration of ghrelin was also reduced in the breast milk of mothers with obese infants. Ghrelin plays a regulatory role in the secretion of growth hormone by the pituitary gland, which, in turn, influences the secretion of various other growth factors, including EGF.

### 4.5. Adipokines

Adipokines, peptide hormones primarily released by adipocytes, play significant roles in regulating metabolic functions within various tissues, including adipose tissue, the liver, the brain, and muscle. They are also found in breast milk and have immunoregulatory properties that help mitigate intestinal inflammation. Examples include adiponectin, which crosses the intestinal barrier and regulates insulin sensitivity while suppressing inflammatory responses, and leptin, which influences infant metabolism, weight regulation, and immune functions like T-cell stimulation [33,141,142,143].

Obesity results in significant alterations in adipose tissue, influencing its size, distribution, cellular compositions, and functions. These changes may manifest as adipocyte hypertrophy, ectopic fat deposition, increased fibrosis, hypoxia, and chronic stress within the adipose tissue. These alterations lead to an unfavorable shift in the release of adipokines, disturbing the body’s overall homeostasis and contributing to obesity-related metabolic complications. These issues encompass the development of insulin resistance, type 2 diabetes, and an elevated susceptibility to cardiovascular disease [144,145,146].

Breast milk delivers these adipokines from mothers to infants, impacting energy intake, growth, and development. There is evidence that breast milk adipokines may alter blood adipokine concentrations in infants, potentially influencing obesity risk and metabolic health in later life. Notably, the presence of these adipokines in breast milk is correlated with maternal factors like BMI and body composition, suggesting a link between maternal health and infant growth [141].

Several articles have demonstrated that the concentration of adipokines in the milk of mothers with obesity was higher than in the milk of mothers with normal body weight [91,92,93]. A single study yielded contrasting outcomes [94].

Zhang’s group [95] study established an inverse relationship between maternal obesity and ghrelin concentration in breast milk. A similar outcome was observed in a 2018 study [93], underscoring that milk from mothers with normal weight contained higher ghrelin levels than from mothers with obesity. In contrast to earlier studies, research by Guler et al. [94] revealed notably elevated levels of ghrelin in the pre-feed breast milk of mothers with obesity compared to mothers with normal weight (*p* = 0.025). Conversely, mothers with normal weight exhibited higher levels of adiponectin in their post-feed breast milk than those with obesity. Higher concentrations of ghrelin in breast milk may have the potential to boost infant food intake, resulting in increased consumption of breast milk and subsequent weight gain in the infants. Fields et al.’s [104] research delved into the determinants of leptin concentration in human milk, highlighting factors such as BMI category and infant gender. The study also brought to light a significant positive link between maternal BMI and leptin concentrations in breast milk. Furthermore, other studies corroborated this discovery, revealing higher leptin concentrations in the milk of obese mothers—up to three times more than in milk from mothers of normal weight. Uysal et al.’s [96] study similarly uncovered a substantial correlation between elevated leptin levels and maternal obesity. Several other studies also confirmed this positive association between maternal obesity and leptin concentration in breast milk [50,92,97,98,99,100,101,102,103,105].

A metagenomic analysis conducted as part of Lemas et al.’s [147] study indicated a link between breast milk leptin and the suppression of genes encoding bacterial proteases. These proteases are associated with increased intestinal permeability seen in inflammatory bowel disease, suggesting that breast milk leptin might indirectly enhance intestinal integrity by inhibiting bacteria that contribute to permeability. Additionally, leptin was found to suppress gene sets related to pyruvate metabolism, including pyruvate kinase, a biomarker for low-grade inflammation in pediatric irritable bowel disease. This implies that leptin’s effects on the infant’s gut microbiome could play a role in reducing inflammation and promoting intestinal health.

The potential impact of adipokines on infant growth and obesity risk raises questions about their role in shaping long-term metabolic health. Adipokines found in breast milk, such as leptin, ghrelin, adiponectin, resistin, and visfatin, could contribute to the early programming of metabolic and immune functions, potentially affecting health outcomes in later stages of life [33,101,141,142,143].

### 4.6. K-Casein

K-casein is a glycoprotein, which is a component of casein. This glycoprotein contains sialic acid residues with a negative charge and functions as a soluble receptor analogous to the surface of epithelial cells. Notably, κ-casein demonstrates inhibitory properties against the adhesion of *Helicobacter pylori* to the human gastric mucosa by mimicking epithelial cell surfaces, effectively acting as a soluble receptor analog [121]. Κ-casein levels in early milk are approximately 0.86 g/L, in transitional milk around 0.80 g/L, and in mature milk about 0.55 g/L [48].

Dyndar et al.’s [87] findings revealed that the concentration of casein in the breast milk of obese mothers was 1.5 times lower compared to milk of mothers with a normal BMI. Further research is needed to investigate whether a reduced concentration of casein can negatively impact the inhibition of *H. pylori* adhesion.

### 4.7. Free Amino Acids

Constituting 5–10% of the total amino acid composition in human milk, free amino acids (FAAs) are a notable component. Particularly during the initial three months of lactation, glutamine and glutamic acid stand out as prominent among the array of free amino acids. Notably, glutamine plays a pertinent role in sustaining the integrity of the gut barrier. It collaborates with growth factors to impact cell signaling pathways associated with the proliferation and differentiation of intestinal cells, along with influencing the expression of tight junctions. These tight junctions, comprised of various proteins, establish a physical barrier between adjacent epithelial cells, thereby maintaining the integrity of the intestines. This barrier effectively prevents the intrusion of pathogens and toxins into the intestinal lumen. Additionally, glutamine’s involvement in glutathione production has been associated with anti-apoptotic properties in intestinal cells [58,148,149].

Research conducted by De Luca et al. [107] revealed that in the mature breast milk of obese mothers, there was a 20% increase in the presence of branched-chain amino acids (BCAA), a specific type of FAA. Nonetheless, the elevated BCAA levels found in breast milk, along with indirect indications of a comparable volume of milk consumed, imply that infants breastfed by obese mothers receive a notably greater BCAA intake compared to those nursed by mothers with normal weight. This increased BCAA intake could potentially influence the metabolism of breastfed infants. This suggests that early-life exposure to high BCAA intake might be linked to a greater risk of metabolic changes later in life [107]. In contrast, it was observed that glutamine and kynurenic acid levels were reduced by approximately 30% in the breast milk of mothers who were classified as obese or overweight six months after childbirth. Consequently, this could lead to an increased risk of future cardio-metabolic issues, disruptions in glucose regulation, and adverse neurological outcomes [59,108].

### 4.8. Polyamines

Polyamines are organic polycations derived from amino acids. Beyond their role in cell growth, polyamines have diverse functions, such as mRNA translation, protection from oxidative damage, and bacterial biofilm formation. In breast milk, polyamines including putrescine, spermidine, and spermine, are believed to contribute to gut and organ development, immune system differentiation, and microbial colonization. They also play a crucial role in intestinal maturation and absorption, and emerging evidence suggests they may even help decrease the risk of allergies. Polyamine concentrations in human milk peak within the first weeks postpartum before gradually declining [150,151,152].

The results of Ali et al.’s [109] study revealed that putrescine and spermidine levels were consistently lower in the breast milk from obese mothers throughout the study period, with statistical significance. The significance of extended periods of reduced polyamine intake remains unclear, primarily due to the absence of established recommendations for ideal or adequate polyamine levels in breast milk or daily intake guidelines for infants [109].

### 4.9. Nucleotides

Nucleotides, often deemed conditionally essential in early life, play vital roles in diverse cellular processes, affecting enzymatic activities and functioning as metabolic mediators. Tissues with high growth rates, like the intestinal epithelium and lymphoid cells, depend on external sources of nucleotides as they have limited ability to synthesize nucleotides. Notably, they contribute significantly to the development, maturation, and repair of the gastrointestinal tract, microbial community, and immune system. Beyond their metabolic functions, nucleotides impact body regulation by enhancing antibody responses, aiding iron absorption, and influencing the synthesis of long-chain polyunsaturated fatty acids. Notably, modified nucleosides possess the ability to impede cell proliferation while triggering apoptosis. For young infants, human milk stands out as the most valuable supplier of these essential nucleotides [45,153,154].

A study by Isganaitis et al. [59] delves into pyrimidine and purine derivatives within breast milk. Orotate reduction of around 25% was observed in milk from overweight–obese mothers, potentially impacting neonatal immunity and metabolic processes. Similarly, purine derivatives such as adenosine monophosphate (AMP) and adenine were increased in overweight–obese mothers’ milk, suggesting potential impacts on adipose tissue function, glucose tolerance, and insulin sensitivity. These findings highlight the intricate interplay between nucleotides, maternal obesity, and neonatal health outcomes.

### 4.10. Osteopontin

Osteopontin (OPN) interacts with cell surface integrins and the CD44 receptor to impact biomineralization, tissue remodeling, and immune regulation. It exerts influence on genes associated with cell proliferation, migration, communication, and survival, particularly those in pathways linked to integrin and CD44 receptor signaling. The presence of OPN-producing epithelial cells and macrophages in the lactating mammary gland suggests that the elevated OPN levels in human milk cells may have a significant impact on the immunological development of breastfed infants. These findings suggest that OPN has a positive impact on the developmental processes of the neonatal intestine and immune system. The concentration of osteopontin is approximately 0.180 g/L in colostrum and 0.138 g/L in mature milk [112].

Maternal pre-pregnancy BMI showed a positive association with OPN levels at 7 and 14 days postpartum [110]. Moreover, correlation analysis revealed a positive association between OPN and several factors, including body weight (kg), bone mineral content (kg), skeletal muscle mass (kg), body fat (kg), and visceral fat area (cm²) [111]. Notably, breast milk OPN concentrations were linked to BMI during lactation. These concentrations were lower in mothers with higher BMIs compared to those with normal BMIs during the lactation period. Specifically, the mean breast milk OPN levels for mothers with normal BMIs, overweight, and obesity during lactation were 156.4 ± 46.2 mg/L, 140.8 ± 61.2 mg/L, and 78.9 ± 28.8 mg/L, respectively. Furthermore, breast milk OPN levels were examined in relation to weight gain during pregnancy. Study participants who gained insufficient weight during pregnancy had OPN levels of 158.2 ± 40.3 mg/L, while mothers with adequate weight gain had levels of 149 ± 60.4 mg/L. Those with excessive weight gain exhibited OPN levels of 119.8 ± 57.4 mg/L [112].

### 4.11. CRP

C-reactive protein (CRP) is a plasma protein classified as an acute-phase protein. Its primary biological role is to defend the host against bacterial pathogens and assist in the removal of apoptotic and necrotic cells. CRP is the fundamental component of the body’s initial innate defense mechanisms [155,156]. If breast milk CRP remains intact during digestion, it might play a role in shaping the composition and balance of the early gut microbiota [113].

Sims et al.’s [98] study showed that CRP levels were elevated in mothers classified as overweight in comparison to those with normal weight. Similar conclusions were reached by Whitaker et al. [113], who discovered that breast milk CRP levels tended to rise as pre-pregnancy BMI increased. Notably, women of normal weight who adhered to recommended weight gain guidelines had considerably lower CRP levels in their breast milk compared to those with obesity or those who exceeded the recommended gestational weight gain guidelines. It remains unclear whether elevated CRP levels have any connection with the growth or body composition of offspring [98], although high CRP concentrations in infants could impact cardiovascular health due to their role in controlling cholesterol levels [157].

### 4.12. Microbiome

The initial gut microbiome in healthy term newborns is characterized by bacterial species like *Escherichia coli*, *Enterococci*, *Streptococci*, and *Clostridia*, followed by anaerobes, particularly the *Bifidobacterium* and *Bacteroides* genera. Within human milk, the microbiome is primarily characterized by the prevalence of *Staphylococcaceae* and *Streptococcaceae*, akin to the microbial composition found on the skin. This resemblance suggests a potential role in shaping the initial bacterial community in the neonatal gut. Remarkably, maternal milk serves as a significant bacterial reservoir for the infant’s intestinal ecosystem, with an estimated intake of 1 × 10^5^ to 1 × 10^7^ bacteria per day for a baby consuming 800 mL/day of milk. Interestingly, consistent breastfeeding fosters a shift towards *Bifidobacteria* and *Bacteroides*, with these bacteria relying on lactose as a key nutritional source [121,131,158].

The breakdown of HMOs by *Bifidobacteria* is particularly advantageous for infants, as it often leads to the production of SCFAs, contributing to a healthier microbiome. The composition of SCFAs in the intestinal tract mirrors the collaborative metabolic interactions among various microbial species because no single bacterial genus can break down all types of substrates and produce all four SCFAs through the fermentation of carbohydrates [159]. This highlights the potential of providing HMOs to support microbial balance. Additionally, BM SIgA may also play a role in establishing bifidobacterial communities in the infant gut. Although SIgA’s procolonization mechanism with *Bifidobacteria* is yet to be fully explored, its interaction may indirectly create a favorable environment for *Bifidobacteria* by inhibiting other species [122]. A study by Yi et al. [8] demonstrated the anti-staphylococcal properties of *Lactobacillus rhamnosus* and *Lactobacillus crispatus* derived from BM. *Lactobacillus* spp. have been observed to inhibit pathogens such as *Shigella* spp., *Salmonella* spp., and *E. coli* by hindering their intestinal adhesion. This reinforces the idea that the BM microbiome not only contributes to gut colonization but also supports protection against harmful pathogens [8].

The research by Collado et al. [89] investigated the composition of breast milk microbiota in mothers with different weight profiles. It was found that overweight mothers had higher levels of *Staphylococcus* group bacteria and lower levels of *Bifidobacterium* group bacteria in their breast milk compared to normal-weight mothers. Surprisingly, *Akkermansia muciniphila* was detected in breast milk despite its typical presence in the infant gut microbiota. This bacterium, known for its ability to degrade intestinal mucus, was associated with proinflammatory signals in breast milk, including higher TNF-α and IFN-γ concentrations and lower concentrations of IL-10 and IL-4 during lactation. The prevalence of *Akkermansia muciniphila* was higher in breast milk from overweight mothers [89]. Moreover, the breast milk from obese mothers had a different and less diverse bacterial community compared to that from normal-weight mothers. Higher maternal BMI was linked to increased numbers of *Lactobacillus* in colostrum and higher numbers of *Staphylococcus* in breast milk 6 months postpartum. Conversely, *Bifidobacterium* levels were lower in the breast milk from obese mothers. The same applies to the BM of mothers with excessive weight gain during pregnancy [114]: infants exclusively breastfed by obese mothers displayed differences in their early microbiome composition, particularly a reduction in Gammaproteobacteria, compared to those breastfed by normal-weight mothers [147].

Another study showed that obese mothers before pregnancy had less *Proteobacteria* and more *Bacteroidetes* in their breast milk compared to overweight and healthy mothers. The same relationship applies to overweight mothers compared to normal-weight mothers. At 3 months postpartum, obese mothers had more *Actinobacteria* in their milk [115].

At the genus level, obese mothers before pregnancy had more *Staphylococcus* and *Corynebacterium* in their breast milk. The same trend with *Corynebacterium* was seen in obese mothers at 3 months postpartum. Overweight mothers had more *Brevundimonas* in their milk compared to healthy mothers, both before pregnancy and at 3 months [115].

When looking at the connection between BMI and maternal glucose tolerance status, *Gemella* was more common among overweight mothers with gestational diabetes compared to healthy-weight mothers. Additionally, *Gemella* was higher in mothers with obesity and impaired glucose tolerance compared to overweight and healthy-weight mothers with impaired glucose tolerance [115]. The gestational diabetes mellitus (GDM) subgroup exhibited a significantly higher prevalence of *Prevotella* [160]. Moreover, when comparing the GDM subgroup with the obesity subgroup, differences in microbial composition were evident, with GDM mothers displaying a lower presence of *Streptococcus* and a higher prevalence of *Xanthobacteraceae* and *Rhizobiaceae*. *Rhizobium*, a specific genus, showed the highest relative abundance in GDM [160].

Transmission of viruses through breast milk is also established and is believed to impact the gut ecosystem of growing infants. Notably, the prevalent viruses in both infant and adult gut environments are bacteriophages, with a particular emphasis on podo-, sipho-, and myoviruses, as well as prophages. These phages are known to have a broad host range, infecting various bacterial species, including *Bacillus*, *Lactobacillus*, *Lactococcus*, *Bacteroides*, *Listonella*, and *Staphylococcus*.

They possess the capacity to either eliminate bacteria or transfer advantageous gene functions, potentially shaping the bacterial community and exerting lasting effects on overall health [161,162].

The investigation of how variations in milk microbiota affect an infant’s gut microbiota and overall health requires further in-depth research [115].

### 4.13. Extracellular Vesicles

Human breast milk contains bacterial-origin extracellular vesicles (EVs) that play a significant role in influencing local immune responses to bacterial challenges. These EVs encompass various types, including apoptotic bodies, microvesicles, and exosomes, and carry a diverse cargo of molecules like mRNA, microRNAs (miRNA), and proteins. Bacteria-derived EVs are thought to contribute to infant gut colonization, immunity, and receptor-mediated interactions with host cells. Milk-derived exosomes have been shown to protect intestinal epithelial cells from oxidative stress, regulating inflammation and cell proliferation, and they have broader implications in immunomodulation and cancer [8,163].

MiRNAs found in breast milk exosomes have a wide range of functions, including immune system development, regulation of immune responses, viral defense, and influence in tissue identity maintenance. These miRNAs are associated with various biological pathways, such as metabolic processes and immunological responses. Notably, miR-155, a key miRNA involved in innate immune system regulation and B- and T-cell maturation, exhibits high expression during the early months of lactation [46]. MiRNAs can also play a role in supporting the survival of leukocytes within the infant’s gastrointestinal system [164].

Breast milk mRNAs and miRNAs can be internalized by cells, potentially impacting protein expression at the infant’s mucosal surface, and thereby affecting the development of the immune system. This suggests the intriguing possibility that breast milk components could alter neonatal immune responses to oral vaccines, respiratory pathogens, and colonization. Overall, breast milk EVs and their content have the potential to shape neonatal immune responses and impact health outcomes [163].

In their pioneering study, Kupsco et al. [116] investigated the extracellular vesicle (EV) miRNA profile of human milk, marking a significant milestone in this field. They identified a total of 1523 miRNAs. Among these, the four miRNAs most notably and inversely associated with BMI were miR-128-3p, miR-130a-3p, miR-574-3p, and miR-6881-5p, with respective fold changes of 0.91, 0.93, 0.92, and 0.94. A study by Shah et al. [117] found that miR-148a and miR-30b in breast milk were lower (30% and 42%, respectively) in abundance in overweight/obese mothers at 1 month after childbirth. miR-148a was associated with lower infant weight, fat mass, and fat-free mass, while miR-30b was linked to higher infant weight, body fat percentage, and fat mass at 1 month. Corresponding findings were obtained by Xi et al. [118]. The concentrations of miRNA-30B, let-7a, and miRNA-378 in colostrum showed a significant negative correlation with maternal prepregnancy BMI (*p* < 0.01). Additionally, in mature milk, let-7a exhibited a negative correlation with maternal weight during the later stages of pregnancy (*p* < 0.05). Another study [119] detected distinct miRNA variations in breast milk-derived extracellular vesicles (bEVs) between mothers of normal weight and those with obesity. This investigation highlighted 19 significantly differentially expressed miRNAs in bEVs of obese mothers, including miR-575 and miR-630, which are linked to adipogenesis and glucose metabolism, potentially influencing breastfed infants.

### 4.14. Leukocytes

Leukocytes in breast milk play a crucial role in providing active immunity to the infant and promoting the development of the infant’s immune system by combating pathogens directly through actions like phagocytosis, producing bioactive substances, assisting in the development of the infant’s immune system, and influencing the infant’s digestive tract environment. Leukocytes in BM have the remarkable ability to survive passage through the infant’s digestive tract and migrate to various parts of the infant’s body, including the blood, lymph nodes, spleen, and liver. Additionally, they may help protect the mother’s mammary gland from infections. The composition of leukocytes in breast milk changes as lactation progresses. Initially, colostrum contains a higher concentration of leukocytes (around 146,000 cells/mL), which decreases in transitional (8–12 days postpartum) and mature milk (26–30 days postpartum) to approximately 27,500 and 23,650 cells/mL, respectively. Leukocyte subsets in breast milk exhibit variability, with major types including myeloid precursors, neutrophils, immature granulocytes, and non-cytotoxic T-cells [164,165,166].

Lymphocytes B, accounting for 34% of colostral lymphocytes, migrate from the mother’s intestinal mucosa to the infant’s, playing a crucial role in maternal immunoprotection. They assist the newborn’s immune system, which cannot yet produce SIgA, in reducing pathogen attachment to the intestinal epithelium [167].

Research by Piñeiro-Salvado et al. [106] found a notable decrease in the fraction of B-lymphocytes in the colostrum of obese mothers compared to lean mothers. Specifically, the obese cohort showed a median percentage of 0.17% B lymphocytes, whereas the lean cohort had 0.41%, indicating a statistically significant difference (*p* = 0.029). However, the proportions of the other nine leukocyte subpopulations in colostrum were similar between the two groups.

## 5. Other Biocomponents of Breast Milk

Breast milk contains additional proteins that influence immunity such as lysozyme, α-lactalbumin, lactoperoxidase, and haptocorrin; however, their variation due to maternal factors, including obesity, has not been definitively established. Lysozyme possesses the remarkable ability to lyse Gram-positive bacteria and Gram-negative bacteria when combined with lactoferrin [78]. Furthermore, lysozyme could potentially protect infants against the risk of intestinal inflammation associated with NEC [48,58]. α-Lactalbumin plays a role in the maturation and development of the infant gut, facilitates the absorption of amino acids, and supports lactose synthesis. Notably, specific polypeptide fragments derived from α-lactalbumin exhibit antimicrobial properties against various pathogens [8,48,168,169]. Lactoperoxidase participates in eliminating both Gram-positive and Gram-negative bacteria and might play a role in enhancing defense against potential infections in the oral and upper gastrointestinal regions [131,168]. Haptocorrin exerts a growth-inhibiting effect on bacteria and serves as a defense mechanism against infections in breastfed infants [131,168]. Due to the protective functions of the mentioned biocomponents, it is necessary to clarify whether they are disrupted by maternal obesity.

## 6. Conclusions

Breast milk is a complex and dynamic fluid that provides a multitude of bioactive components essential for the growth, development, and protection of newborns. The composition of breast milk is influenced by various maternal factors, including BMI, blood pressure, and blood glucose level, and these factors can have significant implications for neonatal health. In recent years, metabolic disorders in mothers have emerged as a significant factor influencing the immune properties of breast milk and subsequently impacting the health of infants. This comprehensive review has shed light on the complex interplay between maternal adiposity and breast milk composition, offering insights into the potential consequences for neonatal immune development and overall well-being.

Maternal metabolic issues introduce alterations in breast milk components that are integral to the infant’s immune defense mechanisms. Shifts in levels of immunoglobulins, cytokines, adipokines, and other bioactive elements in breast milk from obese mothers suggest a potential compromise of the infant’s ability to mount effective immune responses. These immunological changes may contribute to a heightened susceptibility to infections, including gastrointestinal and respiratory illnesses, related complications during early infancy, as well as long-term health risks such as obesity and metabolic disorders.

Reduced levels of key oligosaccharides (2′-fucosyllactos and LNFPI) raise concerns about compromised infection protection and potential effects on infant growth. Indicated changes in monosaccharides suggest implications for the infant’s oxidative stress response, namely the elevated arabinose levels may influence bacterial activity, while increased G6P levels could contribute to enhanced energy sources for infants [59]. The initially elevated IL-6 concentration in colostrum from overweight and obese mothers suggests an impact on inflammatory responses, but its subsequent decrease at 1 month raises questions about the long-term effects on infant growth [90,103]. Furthermore, a metagenomic analysis linking breast milk leptin to the suppression of bacterial protease genes suggests a role in promoting intestinal integrity and reducing inflammation [147]. Additionally, the inhibitory effects of κ-casein against *Helicobacter pylori* adhesion, combined with its reduced concentrations in breast milk from obese mothers, warrant further exploration [87,121]. Amino acid variations, especially increased BCAA, raise concerns about metabolic effects on breastfed infants [107]. Furthermore, alterations in pyrimidine and purine derivatives suggest potential impacts on neonatal immunity, adipose tissue function, glucose tolerance, and insulin sensitivity [59]. On the other hand, the increased presence of *Akkermansia muciniphila* in breast milk from overweight mothers, associated with proinflammatory signals, adds complexity to the relationship between maternal obesity, breast milk microbiota, and neonatal health [89]. However, it is crucial to acknowledge that the field of research in this area is dynamic, with ongoing investigations aimed at unraveling the precise mechanisms linking maternal obesity, commonly accompanied by hypertension and diabetes, with breast milk composition and infant outcomes. The current state of knowledge in this area suggests the necessity for more detailed research, as illustrated in this study.

Although breastfeeding is believed to benefit the newborn, it remains an open question whether these benefits extend seamlessly to mothers grappling with metabolic conditions. In light of these revelations, efforts to address maternal irregularities and promote maternal health can have a profound and lasting impact on the immune protection and overall health of the next generation. The journey toward healthier beginnings for infants born to mothers with obesity is driven by ongoing research and a shared commitment to the brighter, healthier future we aspire to offer the youngest members of our society.

## Figures and Tables

**Table 1:** Alterations in the immunological properties in the milk of obese mothers.

Bioactive Component	Alterations	References	Values
Carbohydrates			
HMOs			
LNFPI	Decrease	Isganaitis et al. [59]	reduction of 60%
LNFPII/III	Increase	Isganaitis et al. [59]	increase of 1.674×
2′-FL	Decrease	Isganaitis et al. [59]	reduction of 40%
	Increase	Lagstrom et al. [60], Wang et al. [61]	increase of 8 nmol/L
Igs			
IgG	Decrease	Dyndar et al. [87]	2.1× lower
2. IgM	Decrease	Dyndar et al. [87]	1.9× lower
3. IgA	Decrease	Dyndar et al. [87]	2.2× lower
4. SIgA	Increase	Fujimori et al. [55]	no specific data
Lactoferrin	Increase	Houghton et al. [88]	increase of 0.36–0.76 mg/mL
	Decrease	Dyndar et al. [87]	1.6× lower
Cytokines			
1. IL-6	Increase	Collado et al. [89]	increase of 18.99 pg/mL (colostrum)
	Decrease	Collado et al. [89]	decrease of 8.9 pg/mL (1-month milk)
2. IL-10	Increase	Collado et al. [89]	increase of 2.55 pg/mL (colostrum)
3. IL-4	Increase	Collado et al. [89]	increase of 2.57 pg/mL (colostrum)
4. TNFα	Increase	Collado et al. [89]	increase of 1.54 pg/mL (colostrum)
	Decrease	Collado et al. [89]	decrease of 0.37 pg/mL (1-month milk)
Growth factors			
1. EGF	Decrease	Khodabakhshi [90]	decrease of 0.002 ng/mL
Adipokines			
1. Adiponectin	Increase	Martin et al. [91], Clark et al. [92]	increase of 3653 pg/mL
		Yu X et al. [93]	
	Decrease	Guler et al. [94]	no specific data
2. Ghrelin	Decrease Increase	Zhang et al. [95], Yu X et al. [93] Guler et al. [94]	decrease of 145.53 pg/mL no specific data
3. Leptin	Increase	Uysal et al. [96], Clark et al. [92], Quinn et al. [97], De Luca et al. [50], Sims et al. [98], Young et al. [99], Savino et al. [100], Chan et al. [101], Kugananthan et al. [102], Fields et al. [103,104], Schuster et al. [105]	96.5% (overweight) and 315.1% (obese) higher; 1.5–2.5 times higher; increase of 830.6 pg/mL; increase of 1.3–2.3 ng/nL; 1.59 log unit increase; increase of 0.006 ± 0.002 ng/mL
Leukocytes			
1. B lymphocytes	Decrease	Piñeiro-Salvado et al. [106]	reduction of 0.24%
Casein	Decrease	Dyndar et al [87]	1.5× lower
FAA	Increase	De Luca et al. [107]	increase of 20%
	Decrease	Bardanzellu et al. [108]	reduce of 30%
Polyamines	Decrease	Ali et al. [109]	decrease of about 10 nmol/dL (spermidine) and 10–30 nmol/dL (putrescine) and depending on the month
Nucleotides			
1. Pyrimidines	Decrease	Isganaitis et al. [59]	reduction of 25%
2. Purines	Increase	Isganaitis et al. [59]	increase of 1.76×
Osteopontin	Increase	Zhu et al. [110], Ruan et al. [111]	no specific data
	Decrease	Aksan et al. [112]	decrease of 16–77.1 mg/L
CRP	Increase	Sims et al. [98], Whitaker et al. [113]	no specific data
Microbiome			
*Staphylococcus*	Increase	Collado et al. [89], Cabrera-Rubio et al. [114], Lemay-Nedjelski et al. [115]	increase of 0.42–0.62 copies/mL
2. *Bifidobacterium*	Decrease	Collado et al. [89], Cabrera-Rubio et al. [114]	decrease of 0.26–0.64 copies/mL
3. *Akkermansia muciniphila*	Increase	Collado et al. [89]	increase of 0.03–0.51 copies/mL
4. *Lactobacillus* 5. *Bacteroidetes* 6. *Proteobacteria* 7. *Actinobacteria* 8. *Corynebacterium* 9. *Brevundimonas*	Increase Increase Decrease Increase Increase Increase	Cabrera-Rubio et al. [114] Lemay-Nedjelski et al. [115] Lemay-Nedjelski et al. [115] Lemay-Nedjelski et al. [115] Lemay-Nedjelski et al. [115] Lemay-Nedjelski et al. [115]	ratio: 0.52; 95% incidence rate ratio: 2.56 incidence rate ratio: 0.62 incidence rate ratio: 2.02 incidence rate ratio: 4.98 incidence rate ratio: 8.72
EVs			
1. miR-128-3p	Decrease	Kupsco et al. [116]	fold changes of 0.91
2. miR-130a-3p	Decrease	Kupsco et al. [116]	fold changes of 0.93
3. miR-574-3p	Decrease	Kupsco et al. [116]	fold changes of 0.092
4. miR-6881-5p	Decrease	Kupsco et al. [116]	fold changes of 0.94
5. miR-148a	Decrease	Shah et al. [117], Xi et al. [118]	lower by 30%, r/F—−0.291/−0.356
6. miR-30b	Decrease	Shah et al. [117]	lower by 42%
7. let-7a	Decrease	Xi et al. [118]	r /F—−0.425/−0.202
8. miRNA-378	Decrease	Xi et al. [118]	r/F—−0.223/−0.335
9. miR-575	Decrease	Cho et al. [119]	log2Fold change = −1.983
10. miR-630	Decrease	Cho et al. [119]	log2Fold change = −1.447

## Data Availability

The data can be shared upon request.
